# An environmental isolate of *Pseudomonas*, 20EI1, reduces *Aspergillus flavus* growth in an iron-dependent manner and alters secondary metabolism

**DOI:** 10.3389/fmicb.2024.1514950

**Published:** 2025-01-20

**Authors:** Elizabeth M. Wyman, W. Scott Grayburn, Matthew K. Gilbert, Matthew D. Lebar, Jessica M. Lohmar, Jeffrey W. Cary, Thomas J. C. Sauters, Antonis Rokas, Ana M. Calvo

**Affiliations:** ^1^Department of Biological Sciences, Northern Illinois University, DeKalb, IL, United States; ^2^Food and Feed Safety Research Unit, USDA/ARS, Southern Regional Research Center, New Orleans, LA, United States; ^3^Department of Biological Sciences and Evolutionary Studies Initiative, Vanderbilt University, Nashville, TN, United States

**Keywords:** *Aspergillus flavus*, *Pseudomonas*, biocontrol, transcriptome, secondary metabolism, aflatoxin, iron, MtFA

## Abstract

**Introduction:**

*Aspergillus flavus* is an opportunistic pathogenic fungus that infects oilseed crops worldwide. When colonizing plants, it produces mycotoxins, including carcinogenic compounds such as aflatoxins. Mycotoxin contamination results in an important economic and health impact. The design of new strategies to control *A. flavus* colonization and mycotoxin contamination is paramount.

**Methods:**

The biocontrol potential of a promising new isolate of *Pseudomonas* spp., 20EI1 against *A. flavus* was assessed using bioassays and microscopy. To further elucidate the nature of this bacterial-fungal interaction, we also performed chemical and transcriptomics analyses.

**Results:**

In the present study, *Pseudomonas* spp., 20EI1 was able to reduce the growth of *A. flavus*. Furthermore, we determined that this growth inhibition is iron-dependent. In addition, *Pseudomonas* 20EI1 reduced or blocked the production of aflatoxin, as well as cyclopiazonic acid and kojic acid. Expression of iron-related genes was altered in the presence of the bacteria and genes involved in the production of aflatoxin were down-regulated. Iron supplementation partially reestablished their expression. Expression of other secondary metabolite (SM) genes was also reduced by the bacteria, including genes of clusters involved in cyclopiazonic acid, kojic acid and imizoquin biosynthesis, while genes of the cluster corresponding to aspergillicin, a siderophore, were upregulated. Interestingly, the global SM regulatory gene *mtfA* was significantly upregulated by 20EI1, which could have contributed to the observed alterations in SM.

**Discussion:**

Our results suggest that *Pseudomonas* 20EI1 is a promising biocontrol against *A. flavus*, and provide further insight into this iron-dependent bacterial-fungal interaction affecting the expression of numerous genes, among them those involved in SM.

## 1 Introduction

*Aspergillus flavus* is an opportunistic phytopathogen found colonizing oil seed crops such as peanut, corn, sorghum, tree nuts and cotton (Robens and Cardwell, 2003). This fungus efficiently disseminates by forming air-borne conidia present on specialized structures denominated conidiophores. The fungus also forms resistant structures termed sclerotia, which allows survival under harsh conditions for several years (Horn et al., 2014). Sclerotia germinate to form additional hyphae or conidia, further facilitating further spreading in the field (Hedayati et al., 2007; Amaike and Keller, 2011).

Upon colonization of the plant substrate, *A. flavus* synthesizes several mycotoxins, including aflatoxins (AFs) (Cary et al., 2015; Bhatnagar et al., 2018). Exposure to mycotoxins can have lasting health impacts. AF is classified as a Group-1 carcinogen by the International Agency for Research on Cancer (Kale et al., 2008). AFB_1_ has been linked to liver, lung, and kidney cancers, immunosuppression, aflatoxicosis and growth retardation in children (Williams et al., 2004; Amaike and Keller, 2011; Hyde et al., 2018; Kinyungu et al., 2019). AF contamination is a worldwide problem (Mamo et al., 2022). Numerous countries enforce strict AF limits in crops to reduce health impacts, leading to major economic losses, as contaminated crops are often destroyed or reduced in value. Annually contaminated crops account for $1 billion US in economic losses (Robens and Cardwell, 2003; Wu et al., 2008; Cary et al., 2019); AF contamination caused the loss of approximately 25% of the world’s food crops yearly (WHO, 2018). Some developing countries do not have legislation to control AF limits, and 4.5 billion people are at risk of ingesting AF (Drott et al., 2021). *A. flavus* also produces other toxic secondary metabolites (SMs), such as cyclopiazonic acid (CPA), which can also result in health impacts. CPA inhibits mammalian Ca^2+^-ATPases and disrupts normal intracellular calcium flux (Chang and Ehrlich, 2011). Normal calcium flux is vital to cellular life and its disruption leads to cell death. Usually, toxicity can occur through ingestion and exposure to contaminated food and feed products (Norred et al., 1988; Dorner et al., 1994; Oliveira et al., 2008; Chang and Ehrlich, 2011).

Current control methods for reducing mycotoxin crop contamination are still limited. Azole fungicides are applied abundantly to crops, leading to the acquisition of antifungal resistance through environmental routes (Kleinkauf et al., 2013) that could reduce their efficacy. Furthermore, azoles are one of the main forms of antifungal drugs used in the medical field, and therefore their extensive use in agriculture could reduce their efficacy when treating fungal infections in humans and animals (Snelders et al., 2009; Chowdhary et al., 2014; Ahangarkani et al., 2020; Barber et al., 2020). Thus, it is important to develop new methodologies, including biocontrols, to reduce fungal growth and dissemination, as well as mycotoxin contamination of crops. Recent studies have examined the impact of non-aflatoxigenic strains being introduced to outcompete toxigenic species (Amaike and Keller, 2011; Kinyungu et al., 2019; Moore, 2022), and although applying an non-aflatoxigenic biocontrol to corn post-harvest shows a decrease in *A. flavus* contamination (Kinyungu et al., 2019), many regions remain infected by *A. flavus* as non-aflatoxigenic strains are not adapted to all environmental conditions.

Thus, finding new strategies to protect crops from fungal infections and mycotoxin contamination is key to prevent their negative impact. Previous studies showed that compounds of plant origin present inhibitory activity against pathogens of plants and animals, including humans (Radha et al., 2024). Also, some nanomaterials with antifungal effect, such as copper nanoparticles (Qin et al., 2023), have been biologically synthesized in fungi (Gaba et al., 2022). These mycogenic copper oxide nanoparticles also showed antifungal activity. Additionally, bacteria and their bioactive metabolites have the potential to be utilized as biocontrol agents against fungal plant pathogens (Veliz et al., 2017; Chalivendra and Ham, 2019; Legein et al., 2020). The use of bacterial biocontrol agents presents certain advantages over other methods. For instance, bacteria can adapt to many environments and often outcompete pathogens for resources and nutrients due to being better acclimatized (Elnahal et al., 2022), and even improve plant health by increasing the plant’s ability to endure abiotic and biotic stresses (Elnahal et al., 2022). Previous reports have indicated the potential of bacteria and bacterial compounds against numerous fungal species. For example, antimicrobials from *Bacillus* species have been shown to reduce *Fusarium* spp. infection (Gong et al., 2015; Alberts et al., 2016; Palazzini et al., 2016). *Bacillus* spp. (including the recategorized *Lysinibacillus* spp.) have also been used against *A. flavus* and *A. parasiticus*, negatively affecting fungal growth as well as aflatoxin production (Raksha Rao et al., 2017; Xia et al., 2017; Chalivendra et al., 2018; Shu et al., 2018). Additionally, *Sporosarcina* sp., *Pantoea* sp., *Lysinibacillus fusiformis* and *Staphylococcus warneri* have been utilized to degrade aflatoxins using *in vitro* assays (Adebo et al., 2016; Xie et al., 2019). *A. flavus* growth and expression of aflatoxin biosynthetic genes were reduced in *Priestia megaterium* and *Wickerhamomyces anomalus* co-cultures (Kong et al., 2014; Hua et al., 2019; Gupta et al., 2020). Strains of *B. subtilis* and *Vibrio gazogenes* were also shown to inhibit expression of the AF genes *aflD* and *aflR* (Al-Saad et al., 2016; Kandel et al., 2022). However, the factors causing this inhibition remains largely unknown.

Various strains of *Pseudomonas fluorescens* have demonstrated its potential as biocontrol (Kerr, 1999; Nagarajkumar et al., 2004; Barahona et al., 2011). For example, *P. fluorescens* causes fungal growth inhibition in *Fusarium oxysporum*, *Macrophomina phaseolina*, *Magnaporthe grisea*, *Cercosporidium personatum*, and *Rhizoctonia solan*i (Ganeshan and Manoj Kumar, 2005; Gull and Hafeez, 2012). *F. verticillioides* had reduced incidence when maize was treated with *P. fluorescens* (Chandra Nayaka et al., 2009). *P. fluorescens* reduces cell wall degradation in radish and carnation by *F. oxysporum* (Ganeshan and Manoj Kumar, 2005). Also, *P. fluorescens*-treated rice seeds showed a reduction in *Xanthomonas oryzae* colonization (Ganeshan and Manoj Kumar, 2005). In addition, rice blast incidence from *M. grisea* was reduced by *P. fluorescens*. Strains of *P. fluorescens* have shown to inhibit mycelial growth of sheath blight fungus *R. solani* (Ganeshan and Manoj Kumar, 2005; Gull and Hafeez, 2012). Several factors could be involved in the inhibition of fungal growth caused by *P. fluorescens*, such as the production of antifungal metabolites (Chin-A-Woeng et al., 2000; Dutta et al., 2020). For example, 2,4-diacetylphloroglucinol, phenazines, pyoluteorin, pyoverdin, pyrrolnitrin, siderophores, lipopeptides and hydrogen cyanide are all SMs produced by *P. fluorescens* that have antimicrobial and antifungal properties. Many of these metabolites have been found to inhibit hyphal formation, hydrolyze the fungal cell wall, interfere with cell membrane permeability and nutrient acquisition in fungal phytopathogens (Ganeshan and Manoj Kumar, 2005; Gull and Hafeez, 2012; Veliz et al., 2017; Legein et al., 2020; Kandel et al., 2022).

Other *Pseudomonas* species have also been evaluated as possible biocontrols, including *P. aeruginosa*, *P. corrugata*, *P. chlororaphis*, and *P. protegens*. *P. aeruginosa* is a well-studied biocontrol against fungi. It produces a variety of antifungal SMs including phenazine, hydrogen cyanide, siderophores, pyoverdine, pyochelin and rhamnolipid-type biosurfactants (Höfte, 2021). *Pseudomonas corrugata* is another biocontrol that inhibits growth of filamentous fungi (Höfte, 2021). For instance, *P. corrugata* inhibited fungal growth of *Gaeumannomycis graminis* var. *tritici* in wheat (Harrison et al., 1993), and reduces disease on strawberry and tomato by *F. oxysporum* and *Phytophthora cactorum* (Barahona et al., 2011). *Pseudomonas chlororaphis* is yet another biocontrol against filamentous fungi. *P. chlororaphis* has been shown to inhibit mycelial growth in *F. graminearum*, *Colletotrichum gloeosporioides*, and *Botrytis cinerea* (Huang et al., 2018). *P. chlororaphis* also induces resistance in cucumber toward *Corynespora cassicola* (Kim et al., 2004). *P. protegens* represses growth of *R. solani* and *P. ultimum* on cotton (Howell, 1979; Howell and Stipanovic, 1980). This organism also inhibits growth of *F. oxysporum in vitro* (Takeuchi et al., 2014). Additionally, *P. protegens* reduced incidence of *P. ultimum* in cucumber (Takeuchi and Someya, 2019).

As mentioned above, *Pseudomonas* species are important biocontrols antagonistic to fungal phytopathogens. The present study provides further insight into the mechanism of action of a new isolate of *Pseudomonas*, 20EI1, against *A. flavus*, and its possible dependency on iron acquisition, using both chemical and transcriptomics approaches. An in-depth understanding of the bacterial-fungal interaction is essential for the development of a successful control strategy to decrease *A. flavus* phytopathogenicity and mycotoxin contamination.

## 2 Materials and methods

### 2.1 Isolation and identification of *Pseudomonas* 20EI1

The environmental isolate 20EI1 was isolated from a lagoon in DeKalb, Illinois, United States, at a depth of 0.5 m. The 20EI1 genomic DNA was extracted and used as template for PCR. The amplification was carried out using primers 515F-Y (GTGYCAGCMGCCGCGGTAA) (Parada et al., 2016) and DG74 (AGGAGGTGATCCAACCGCA) (Greisen et al., 1994). These primers amplified 16S rRNA. The PCR product was sequenced and a Blastn analysis revealed a match with the genus *Pseudomonas* (Altschul et al., 1990; Camacho et al., 2009).

### 2.2 Microbial strains, culture conditions and assessment of fungal and bacterial growth

The new isolate of *Pseudomonas* 20EI1, and *A. flavus* AF70 wild-type strain were used in this study. Bacteria were inoculated and grown in overnight cultures. Bacterial cells and fungal spores were co-cultured on solid PDA or modified Czapek-Dox (CZ, 0.5% glucose, 0.1% yeast extract, pH 7.3 ± 0.3), conducive to the growth of both organisms as indicated. Top agarose (0.7%) was inoculated with 0.3 mL overnight bacterial culture in 4.7 mL top agarose for a total of 5 mL and poured onto 20 mL solid medium plates. Then, 2 μL of fungal spore suspension (in concentration of 10^6^ spores/mL) was point-inoculated at the center of the plates. In a separate experiment, plates were top-agarose inoculated with 10^6^ spores/mL of *A. flavus*. Then 2 μL of bacterial suspension was point-inoculated on these plates. Plates were incubated at 30°C in the dark. The experiments were carried out in triplicate.

Both organisms were also co-cultured in liquid PDB medium (adjusted pH to 7.3 ± 0.3). In this case, in order to test whether antifungal activity was iron-dependent, liquid cultures were set with and without iron supplementation (200 μg/mL FeCl_3_). Controls of fungal cultures without the bacterial treatment and bacterial cultures without the fungus were included. Inoculation was carried out with 24 h bacterial and fungal seed cultures growing in PDB medium at 30°C and 150 rpm shaking conditions. Three mL of bacterial culture and about 0.12 g of mycelia were added to fresh PDB. Filter sterilized FeCl_3_ solution was then added to the cultures. Cultures were then grown for 48 h in a shaker incubator at 150 rpm and 30°C. The experiments included three replicates. Separation of mycelia from the bacteria was carried out by filtration using miracloth. Mycelia was collected to measure dry weight. Bacterial OD_600_ readings were acquired to assess bacterial growth using a Denovix DS-11 Spectrophotometer (Denovix, CT).

### 2.3 Scanning electron microscopy

To obtain scanning electron microscopy (SEM) micrographs, liquid co-cultures and monoculture controls were grown as previously described. Liquid cultures were aliquoted into 1.5 mL Eppendorf tubes. Samples underwent fixation as described by Simon et al. (2015). Briefly, samples were fixed for 1 h with aldehyde fixative, followed by 1 h in osmium fixative. Samples were sequentially dehydrated using ethanol at 30, 50 and 70% concentrations, for 20 min each and then changed to 100% for 10 min, three times. A critical point dryer (Tousimis CPD) was used to replace ethanol with CO_2_. Samples were then sputter coated with gold using Denton Desk IV Sputter Coater. Images were taken using a Hitachi S3400N SEM (NUANCE, Evanston, IL) at the NUANCE facility at Northwestern University.

### 2.4 Chemical analysis

Liquid cultures were set as described above. Five mL of supernatant was collected into a 50 mL falcon tube. Five mL of chloroform was added and extracted overnight. The bottom organic layer was collected and put into a clean beaker and allowed to evaporate. Once evaporated the extract was collected with chloroform and spotted on a TLC plate to detect AF. The plate was run in a solvent system of 85 chloroform:15 acetone (v/v) for 15 min. After 15 min the plate was sprayed with 12.5% AlCl_3_ and baked at 80°C for 10 min. The TLC plate was then visualized and imaged with a UV light.

In a separate experiment, broth cultures (50 mL) were filtered, the filtrates lyophilized (Freezemobile, SP Scientific), and extracted with 20 mL 80% acetonitrile: 19.5% water: 0.5% formic acid on a shaker (200 rpm) for 2 h. Portions of the filtered mycelia (0.15–0.2 g) were also lyophilized then extracted with 1 mL methanol on a shaker (200 rpm) overnight. The extracts were centrifuged to pellet the particulates, and the supernatant was analyzed using a Waters ACQUITY UPLC system (1 μL injections, 40% methanol in water isocratic solvent system on a BEH C18 1.7 μm, 2.1 mm × 50 mm column) using fluorescence detection (Ex = 365 nm, Em = 440 nm). Samples were diluted if the aflatoxin signal saturated the detector. Analytical standards (Sigma-Aldrich, St. Louis, MO, United States) were used to identify and quantify aflatoxins: aflatoxin B_1_ (AFB_1_); aflatoxin B_2_ (AFB_2_). Aflatoxin content was expressed in ppb (ng AF/mL broth or ng AF/g mycelia).

CPA and other metabolites in the extracts were analyzed on a Waters Acquity UPLC system coupled to a Waters Xevo G2 XS QTOF mass spectrometer. Extract injections (1 μL) were separated on a Waters BEH C18 1.7 μm, 2.1 × 50 mm column with the following gradient solvent system: (0.5 mL/min, solvent A: 0.1% formic acid in water; solvent B: 0.1% formic acid in acetonitrile): 5% B (0–1.25 min), gradient to 25% B (1.25–1.5 min), gradient to 100% B (1.5–5.0 min), 100% B (5.0–7.5 min), then column equilibration to 5% B (7.6–10.1 min). The Z-spray ionization source was run in ESI^+^ mode using MassLynx 4.2 software with the following settings: source temperature: 100°C, desolvation temperature: 250°C, desolvation gas flow: 600 L/h, cone gas flow: 50 L/h, capillary voltage: 3.0 kV, sampling cone voltage: 40 V. Analyses were performed in sensitivity and continuum mode, with a mass range of *m*/*z* 50–1,200 and a scan time of 0.1 s. A data-independent acquisition method with elevated collision energy (MS^E^) was used with 6 eV low energy and a high energy ramp from 15–45 eV. Data were analyzed on Waters UNIFI 1.9.4 software and quantified using “Quantify Assay Tof 2D” analysis method with lock mass corrected by UNIFI. CPA was purchased from Sigma-Aldrich (St. Louis, MO, United States) and used for quantification. Aflatrems and aflavinine standards were kind gifts from James Gloer (University of Iowa). Toxin content was expressed in ppb (ng toxin/mL broth or ng toxin/g mycelia).

Kojic acid (KA) analyses were conducted on the Waters Acquity UPLC system coupled to a Waters Xevo G2 XS QTOF system with the same mass spectrometer settings that was used for CPA analysis. The filtered broth culture extracts were diluted 10-fold with LC/MS grade water. Injections (1 μL) were separated on a Waters BEH C18 1.7 μm, 2.1 × 50 mm column with the following solvent system: 0.5 mL/min, solvent A: 0.1% formic acid in water; solvent B: 0.1% formic acid in acetonitrile; pump settings: isocratic at 100% A for 2.5 min (0–2.5 min), gradient to 95% A over 0.5 min (2.5–3.0 min), gradient to 80% A over 1.0 min (3.0–4.0 min), isocratic at 80% A for 1 min (4.0–5.0 min) then column equilibration to 100% A for 2.5 min (5.0–7.5 min). Mass data were collected for the first 2 min of the 7.5 min run, imported into Waters UNIFI 1.9.4 software, and quantified using “Quantify Assay Tof 2D” analysis method with lock mass corrected by UNIFI. Kojic acid was purchased from Sigma-Aldrich (St. Louis, MO, United States) and used for quantification. KA content was expressed in ppb (ng KA/mL broth).

### 2.5 Transcriptome analysis

#### 2.5.1 RNA preparation and sequencing

Liquid co-cultures of *P. fluorescens* and *A. flavus* and their respective monoculture controls were carried out as described in the section above. The amount of iron used was also 200 μg/mL FeCl_3_. Control cultures without iron supplementation were also included. Fungal mycelia were collected from these cultures for RNA extraction. Bacterial and fungal biomass from co-cultures were separated before RNA extraction. To separate bacteria from mycelia, the samples were filtered using miracloth. After that, samples were frozen in liquid nitrogen and stored at −80°C. RNA extraction was carried out as follows: For extraction of RNA from fungal samples, a Maxwell RSC Plant RNA Kit was used following the manufacturer’s directions. Briefly, 20–50 mg of mycelium was placed in a bead tube. 1-T/Homogenization solution was added to the tube and bullet-blended. This step was repeated and then centrifuged at maximum speed for 2 min. The homogenate was then transferred to a microcentrifuge tube and lysis buffer added. Vortexed for 15 s and incubated at room temperature for 10 min. Samples were centrifuged and supernatant was transferred to wells. DNase was added to a separate well. Nuclease free water was added to the bottom of each elution tube.

RNA quality and quantity were determined using Qubit fluorometer (Invitrogen) and analyzed for integrity using Agilent 4200 TapeStation. RNA libraries were prepared using CORALL Total RNA-seq Library Prep Kit with Unique Dual Indices with RiboCop HMRv2 rRNA Depletion Kit (Lexogen). After cDNA synthesis, the 3′ ends of first-strand cDNA fragments were ligated with a linker containing Illumina-compatible P5 sequences and Unique Molecular Identifiers (UMIs). During the following steps of second strand cDNA synthesis and ds cDNA amplification, i7 and i5 indices as well as complete adapter sequences required for cluster generation were added. The number of PCR amplification cycles was 14 as determined by qPCR using a small pre-amplification library aliquot for each individual sample.

Final amplified libraries were purified and quantified, and average fragment sizes were examed by gel electrophoresis using 4200 TapeStation and D5000 Screen Tape (Agilent). The concentration of the final library pool was confirmed by qPCR and the pool was subjected to test sequencing on MiniSeq instrument (Illumina) in order to check sequencing efficiencies and adjust accordingly proportions of individual libraries. Sequencing was carried out on NovaSeq X 10B (Illumina), 2/150 bp reads.

#### 2.5.2 RNAseq read alignment and differential expression calculation

RNAseq reads were quality checked before and after filtering with Trimmomatic (v0.39) (Bolger et al., 2014) using FastQC (v0.12.1, http://www.bioinformatics.babraham.ac.uk/projects/fastqc/). Each sample was aligned to the NRRL3357 reference (GCA_014117465.1) using STAR (v2.711) (Dobin et al., 2013). Read counts per strain were calculated using HTseq (v2.0.5) (Anders et al., 2014). For all software, default settings were used unless otherwise specified.

Using DESEQ2 (v1.42.0) (Love et al., 2014), differential expression was calculated using a multifactored approach. Each treatment group (fungus only, fungus + iron, fungus + bacteria, and fungus +bacteria + iron) had three replicates used to calculate differential expression. The effects of iron and bacteria were determined using the respective single factor treatment groups (“~iron” and “~bacteria”). Using the formula “~bacteria + iron + bacteria:iron” the combined effects of bacteria and iron were determined using all of the treatment groups. Differential expression results were shrunk using the “ashr” shrinkage estimator. Downstream visualizations and analyses were performed using R (v4.4.1, http://www.r-project.org/).

#### 2.5.3 Functional annotation and referencing

GO terms and protein function were determined by running Interproscan (v5.61-93.0) (Jones et al., 2014) on the NCBI reference GCA_014117465.1 using default parameters. For comparison with prior datasets and previously determined genes in biosynthetic gene clusters (BGC), reciprocal DIAMOND (v2.1.6) (Buchfink et al., 2021) BLAST was used between the current NCBI reference (GCA_014117465.1) and an older version (GCF_000006275.2). Hits with the highest bitscore were retained resulting in 11,278 matches. Any genes of interest identified were evaluated on the percent identity (>70%) for reporting.

### 2.6 Statistical analysis

ANOVA tests were used followed by a Tukey’s honest significant difference test, unless otherwise indicated. This *post hoc* test allowed for multiple comparisons to confirm where differences occurred between treatments. GraphPad Prism (version 10.3.1 (509)) was the software used to carry out the statistical analyses.

## 3 Results

### 3.1 Co-culture assays

**Figure 1:**
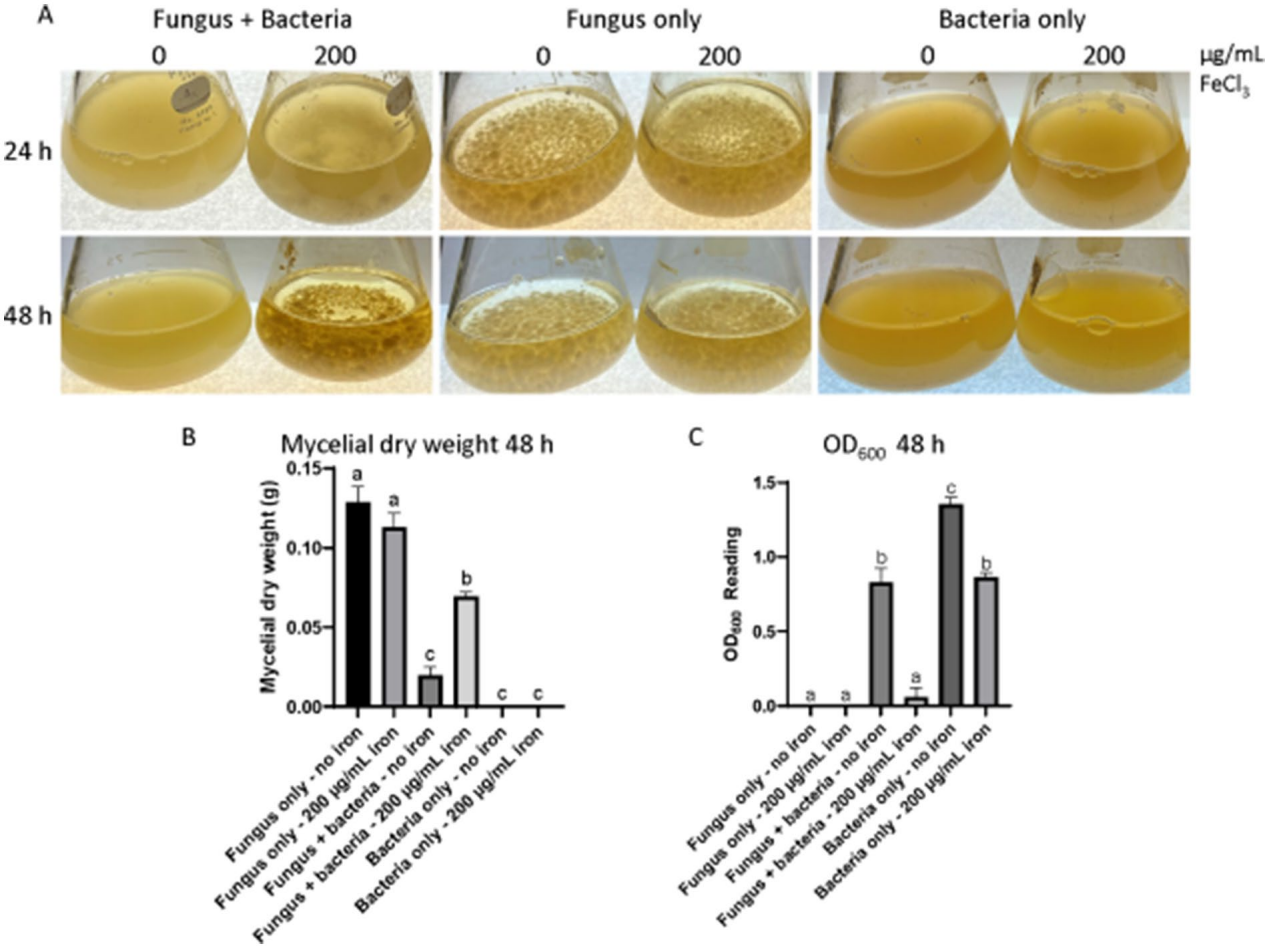
Liquid co-culture assay of *Pseudomonas* 20EI1 and *Aspergillus flavus*. **(A)** *A. flavus* with and without bacterial and iron treatment grown at 30°C for 48 h at 150 rpm. Both organisms were co-cultured in PDB. Controls of fungal cultures without the bacterial treatment and bacterial cultures without the fungus were included. Inoculation was carried out with 24 h bacterial and fungal seed cultures growing in PDB at 30°C and 150 rpm shaking conditions. Filter sterile liquid FeCl_3_ was then added to the corresponding cultures. The experiments included three replicates. **(B)** Dry weight of mycelia collected from cultures in **(A)**. **(C)** Bacterial OD_600_ readings acquired to assess bacterial growth.

Fungal growth inhibition was observed in solid and liquid cultures of *A. flavus* AF70 incubated in the presence of the 20EI1 bacteria (Supplementary Figures S1, S2; Figure 1), the most striking results were detected in solid cultures, where 20EI1 inhibited the formation of *A. flavus* colonies (Supplementary Figure S1B). Additionally, in co-culture studies with a fungal lawn, a growth inhibition halo was observed around 20EI1 (Supplementary Figure S2), whereas this effect was not detected when the *E. coli* control was used or in the absence of bacteria (Supplementary Figure S2C). In liquid cultures, measurement of mycelial dry weight indicated reduction of fungal growth in the co-culture compared to that of the fungus-only control (Figure 1B). Interestingly, fungal growth inhibition was partially lost in liquid co-cultures supplemented with iron (Figure 1A); *A. flavus* growth was reduced 84.6% in co-culture without iron, and 45.9% when in co-culture with iron. In addition, OD_600_ readings indicated that bacterial growth was reduced in the co-cultures with iron (Figure 1C). Addition of iron did not affect the growth of *A. flavus* or *Pseudomonas* 20EI1 when cultured individually.

### 3.2 Scanning electron microscopy of *Pseudomonas* 20EI1 attachment to *Aspergillus flavus* hyphae

**Figure 2:**
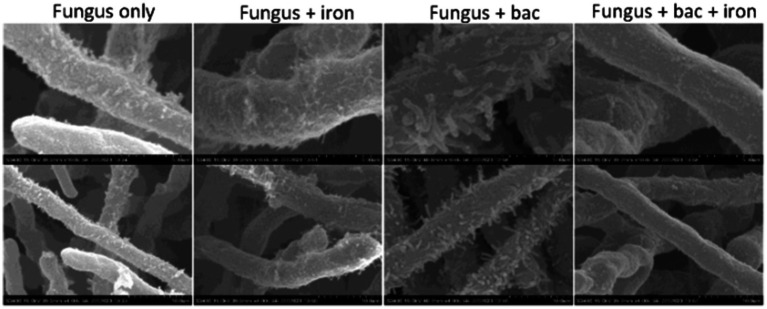
SEM micrographs of *Aspergillus flavus* and *Pseudomonas* 20EI1. Mycelia collected from liquid cultures was fixed using aldehyde and osmium. Samples were sequentially dehydrated using ethanol, critical point dried to replace ethanol with CO_2_ and sputter coated with gold. Micrographs in the top row were taken with ×10k magnification, and those in the bottom row were taken with ×4k magnification.

Some *Pseudomonas* species, such as *P. fluorescens*, has been found to damage the hyphae of *A. flavus* in previous studies (Akocak et al., 2015), we investigated whether 20EI1 had a similar effect. *Pseudomonas* 20EI1 presence in the co-cultures did not result in hyphal leakage, however, a strong attachment of the bacteria to the hyphae was observed (Figure 2). This is not detected in the co-cultures of fungus and bacteria with supplemented iron.

### 3.3 *Aspergillus flavus* secondary metabolism is altered by *Pseudomonas* 20EI1 and iron

**Figure 3:**
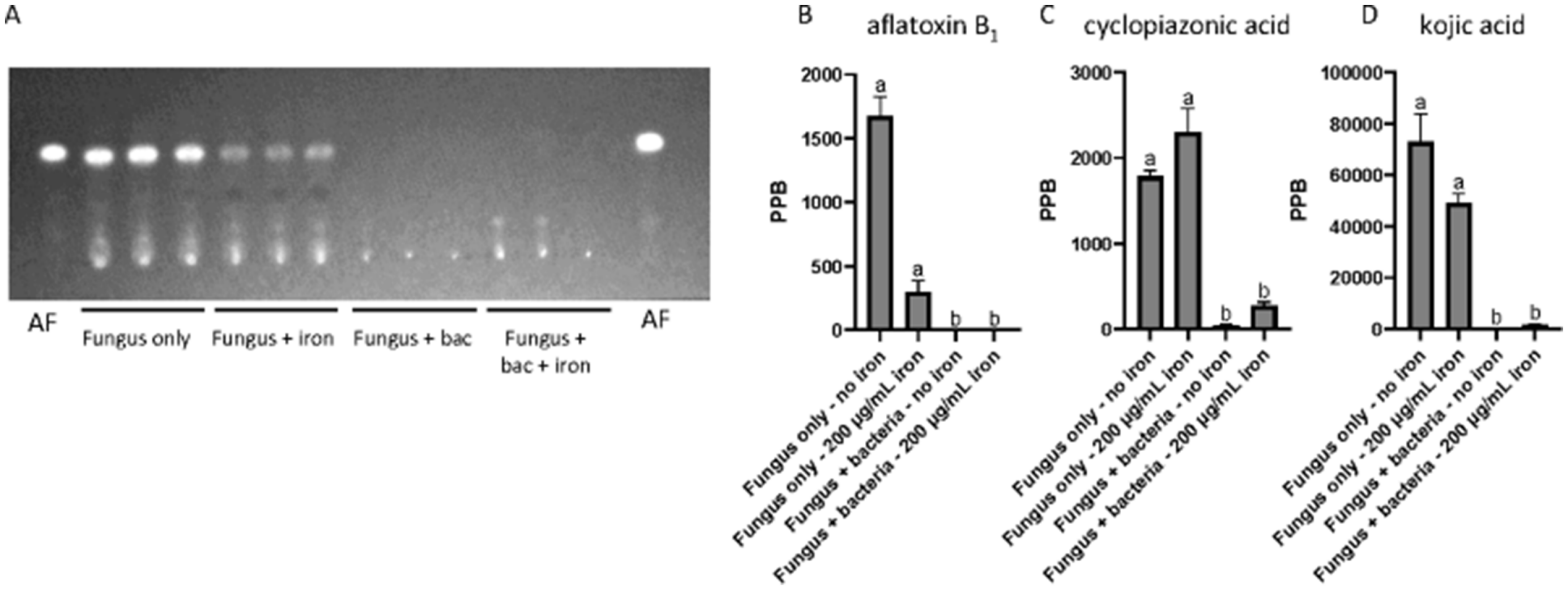
Chemical analyses of *Pseudomonas* 20EI1 and *A. flavus* cultures. **(A)** TLC evaluating production of aflatoxin B_1_ (AFB_1_—indicated as AF in this panel) in liquid cultures. Supernatant was collected and extracted with an equal amount of chloroform. Extracts were spotted on a TLC silica plate and separate in an 85 chloroform:15 acetone (v/v) solvent system. **(B–D)** UPLC quantification of aflatoxin B_1_ **(B)**, cyclopiazonic acid **(C)** and kojic acid **(D)**. Conditions for UPLC analysis were as described in the materials and methods section.

Our chemical analysis indicated that *Pseudomonas* 20EI1 and iron impacted production of SMs in *A. flavus*. TLC analysis of culture supernatant extracts revealed that AF production was reduced in fungal cultures with added iron. Furthermore, the presence of *Pseudomonas* 20EI1 in the fungal culture nearly blocked aflatoxin biosynthesis in this fungus in both supplemented and non-supplemented iron cultures (Figure 3A). UPLC results confirmed these findings, showing a similar pattern of AF production as that shown in the TLC analysis (Figures 3A,B), with a 99.8% reduction in the fungus and bacteria culture. Addition of iron to the co-culture did not rescue AF production, still showing a 99.7% decrease. In addition, iron supplementation resulted in an 82.2% reduction in AF content in the fungal monoculture (Figure 3B).

The presence of the bacteria also altered production of other SMs. Our analysis revealed that CPA production was dramatically reduced in *A. flavus* cultures challenged with *Pseudomonas* 20EI1, independently of iron supplementation, with a 97.6% reduction in the fungus and bacteria co-culture, and an 84.8% reduction in the fungus, bacteria and iron culture (Figure 3C). Similarly, production of KA was also reduced 99.9% in the *A. flavus* cultures in the presence of the bacteria without iron and 97.9% in the co-culture supplemented with iron (Figure 3D). Addition of iron also reduced KA production in the *A. flavus* culture by 32.9% compared to the control (Figure 3D).

### 3.4 Gene expression is influenced by *Pseudomonas* 20EI1 and iron in *Aspergillus flavus*

**Figure 4:**
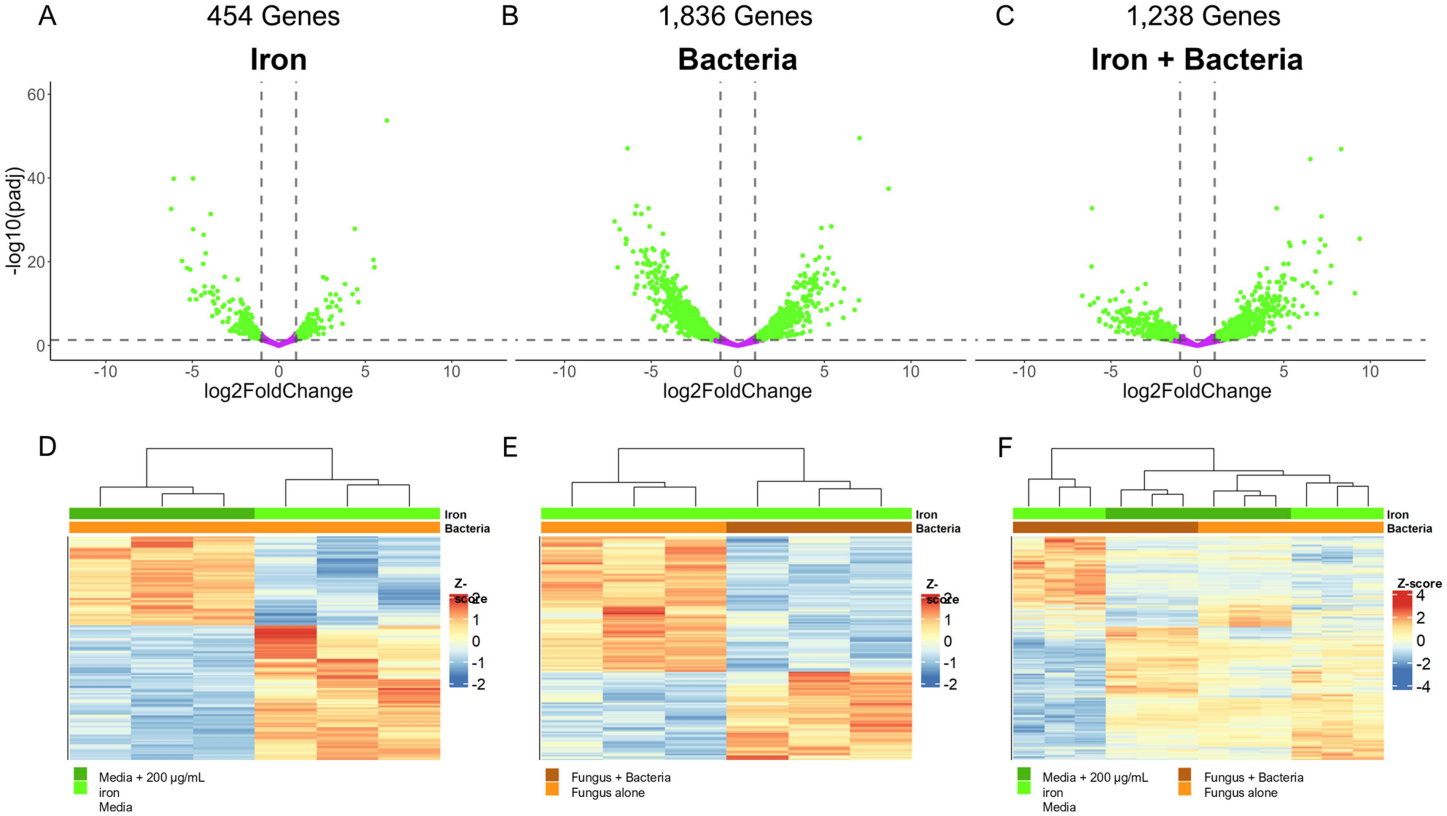
**(A–C)** Volcano plots depicting the significance and fold change of calculated differential expression across all three effect groups: iron **(A)**, bacteria **(B)**, and iron + bacteria **(C)**. Genes significantly, differentially expressed (1 ≥ Log_2_FoldChange ≤ −1, *p*_adj_ ≤ 0.05) are colored green while genes not differentially expressed are colored purple. Dotted lines indicate the significance cut off points. The number of genes differentially expressed are noted above the plots. **(D–F)** Heatmaps depicting the *z*-score of each differentially expressed gene for the effect groups: iron **(D)**, bacteria **(E)**, and iron + bacteria **(F)**. Dendrograms above the plots indicate the sample clustering based on hierarchical clustering of the calculated *z*-scores, and the colored bars beneath the dendrogram indicate the treatment of each sample (green = iron with dark green indicating 200 μg/mL of iron and light green as the control; orange = *Pseudomonas* treatment with dark orange indicating co-culture conditions and light orange as the control).

To understand the effect of *Pseudomonas* 20EI1, iron, and combination of both on *A. flavus* transcriptome we performed RNA-sequencing (Supplementary Table S1). Iron presented the smallest effect on overall gene expression, with 454 genes differentially expressed (1 ≥ Log2FoldChange ≤ −1; *p*_adj_ ≤ 0.05), 176 of which were unique to iron treatment (Figures 4A,D). Due to the relatively small number of differentially expressed genes between the control and iron treatment, there was no enrichment of genes from any functional categories using either gene set enrichment (GSEA) or GO enrichment. Co-culture with *Pseudomonas* 20EI1 had the greatest effect on differential expression, with 1,836 genes differentially expressed, 907 unique to *Pseudomonas* treatment (Figures 4B,E). Based on GSEA, genes involved in copper ion binding and transporter activity were over-represented for genes that are positively expressed, and molybdenum ion binding, heme binding, and iron ion binding were over-represented for genes with reduced expression. The combined effect of *Pseudomonas* and iron resulted in 1,238 genes differentially expressed, with 308 genes unique to the combined effects of *Pseudomonas* and iron (Figures 4C,F). GSEA analysis revealed an overrepresentation of genes annotated in the suppression of branch chained amino acid synthesis that was recapitulated with subsequent GO enrichment along with reduced expression in genes involved in transmembrane transporter activity.

**Figure 5:**
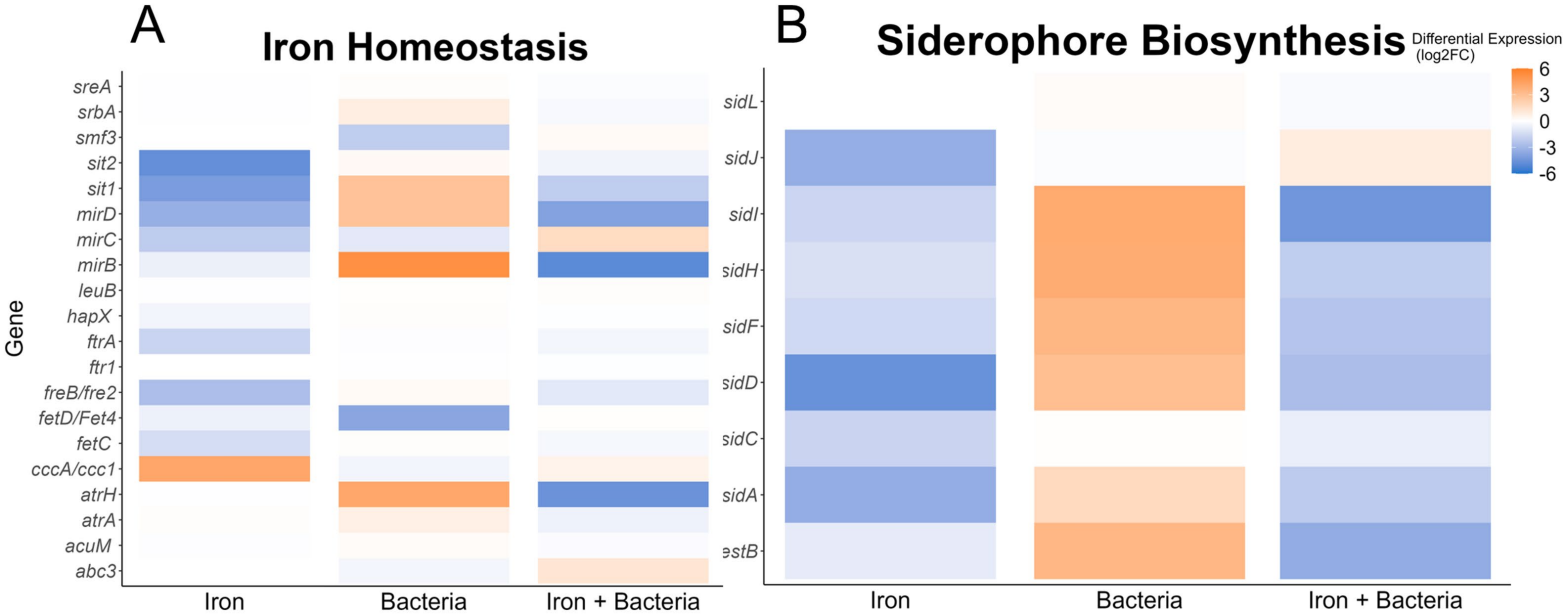
Heatmaps showing differential expression of genes homologous to *Aspergillus fumigatus* iron homeostasis **(A)** or siderophore biosynthetic genes **(B)**. The conditions used to calculate differential expression are indicated along the *x*-axis. Color of the heatmap corresponds to the Log_2_FoldChange of the differential expression.

Our analysis revealed that some *A. flavus* putative homologs to genes with verified roles in iron homeostasis in other fungal species (Misslinger et al., 2021) were differentially expressed in the presence of the bacteria. Specifically, we found that *Pseudomonas* 20EI1 increases the expression (>3-fold) of the siderophore transporters *mirB*, *mirD*, and *sit1*, as well as *atrH* (Figure 5A). Concomitantly, the genes responsible for the biosynthesis of the siderophore triacetylfusarinine C (TAFC), *sidI*, *sidH*, *sidF*, and *sidD*, are all upregulated (>3-fold) in the presence of 20EI1 (Figure 5B). For both, these siderophore transporters and biosynthetic genes, differential expression is reversed when iron is added to the co-culture (Figure 5).

Extending our study, we analyzed the expression changes in genes with functional annotations that involve iron interaction (Supplementary Table S2). Of the 333 genes annotated to interact with iron, 29 were differentially expressed in the presence of iron alone. Of these 29 differentially expressed genes, 13/24 of the predicted iron transporters were differentially expressed (Figure 5; Supplementary Figure S2). The presence of *Pseudomonas* 20EI1 had a greater impact on the 333 iron related genes (Supplementary Table S2), as 70 of them were found differentially expressed in the co-culture, with a similar ratio of downregulated genes to upregulated genes as the iron treated group (44/70 downregulated). Iron transporters were least impacted as only 6 were differentially expressed (4 negatively expressed 2 positively expressed) (Figure 5; Supplementary Figure S2). Interestingly, 39 iron-binding, cytochrome P450 genes were differentially expressed, with the majority (29 genes) displaying reduced expression in the presence of *Pseudomonas*. The combined effect of *Pseudomonas* and added iron resulted in 45/333 iron related genes differentially expressed, with a nearly even split between up and downregulated genes (22 downregulated 23 upregulated). This trend of even expression changes is shared by the annotated iron transporters, with 5 differentially expressed transporters downregulated when both iron and *Pseudomonas* 20EI1 are present (Figure 5; Supplementary Figure S2). Similarly, there is a marked reduction in differentially expressed (only 16 in this treatment) cytochrome P450 genes, and the majority (11/16) of those genes are positively expressed.

**Figure 6:**
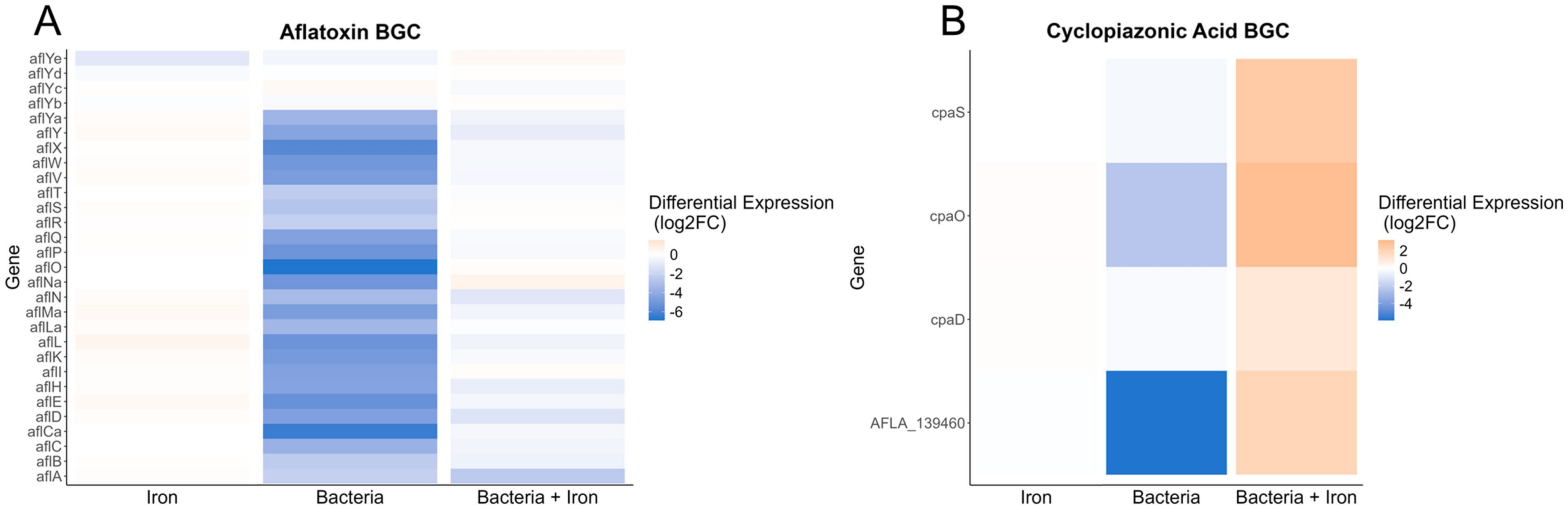
Heatmaps indicating differential expression of all annotated genes within the aflatoxin **(A)** and cyclopiazonic acid **(B)** BGCs. The conditions used to calculate differential expression are indicated along the *x*-axis. Color of the heatmap corresponds to the Log_2_FoldChange of the differential expression.

**Figure 7:**
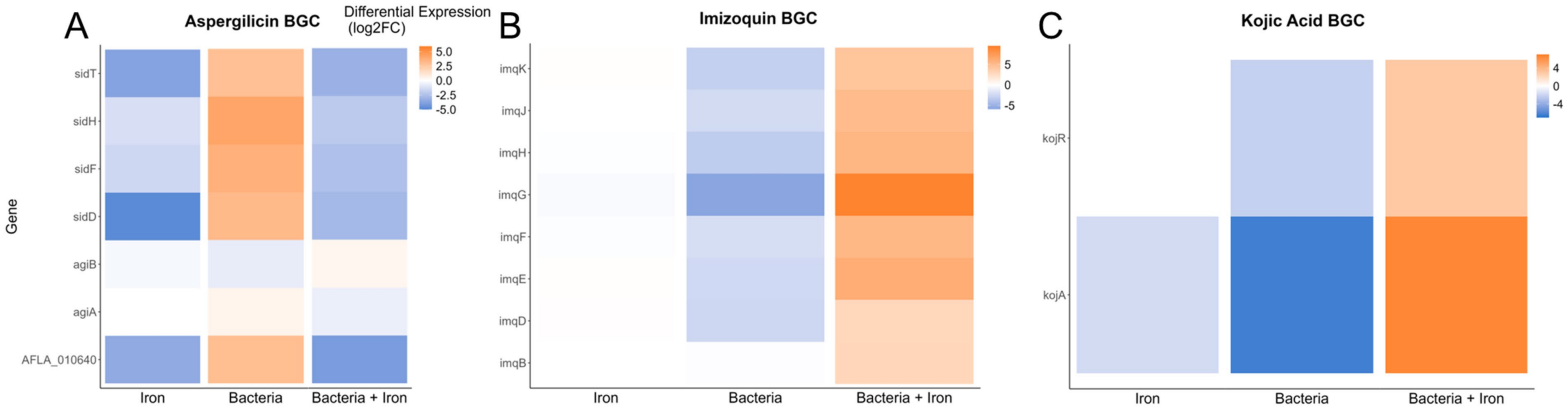
Heatmaps depicting differential expression of all annotated genes within the aspergillicin **(A)**, imizoquin **(B)**, and kojic acid **(C)** BGCs. The conditions used to calculate differential expression are indicated along the *x*-axis. Color of the heatmap corresponds to the Log_2_FoldChange of the differential expression.

Based on the observed impact of *Pseudomonas* and iron on the production of secondary metabolites, such as AF, CPA and kojic acid (Figure 3), we analyzed the expression of *A. flavus* biosynthetic gene clusters (BGCs). Our study revealed that of the more than 60 previously identified BGCs (Georgianna et al., 2010; Cary et al., 2018; Uka et al., 2020), 14 have more than 50% of their genes differentially expressed in one of the three experimental groups (4 for iron, 13 for *Pseudomonas*, and 8 for *Pseudomonas +* iron) (Supplementary Table S3). Notably, *Pseudomonas* 20EI1 overwhelmingly reduces the expression of *A. flavus* BGCs that are differentially expressed. In contrast, the addition of iron during co-culture increases the expression of many of the BGCs with reduced expression in co-culture alone. Specific analysis of BGCs with known products revealed heavily reduced expression of the AF, CPA, kojic acid and imizoquin BGCs (Figures 6A,B, 7B,C). Notably, *Pseudomonas* co-culture increases the expression of genes in the aspergillicin BGC in *A. flavus* (Figure 7A). Adding iron had the opposite effect on all of the BGCs mentioned, other than AF which was only greatly affected by *Pseudomonas* alone (Figure 6A). Iron only has a significant effect (defined by the number of genes differentially expressed) on the aspergillicin BGC, resulting in reduced expression of many genes in this cluster (Figure 6A).

In this study we also examined the expression of a group of global regulatory genes (Supplementary Table S4), including *velvet* genes (Eom et al., 2018), as well as *laeA* (Amaike and Keller, 2009), *hbx1* (Cary et al., 2019), *rtfA* (Lohmar et al., 2019), and *mtfA* (Zhuang et al., 2016). Interestingly, our result revealed that expression of the *A. flavus* putative C_2_H_2_ transcription factor gene *mtfA* was significantly upregulated in the presence of *Pseudomonas* 20EI1.

## 4 Discussion

*Aspergillus flavus* is a plant pathogen that impacts oil seed crops, such as corn, peanuts, tree nuts and sorghum. Current treatments to reduce *A. flavus* colonization and AF contamination of crops are still limited, and resistance to antifungal chemicals is emerging (Kleinkauf et al., 2013). Biocontrol methods have reached certain success, for example the use of non-aflatoxigenic *A. flavus* strains in the field (Cotty, 1990). In addition, treatments with bacteria and bacterial metabolites present promising potential as controls against fungal growth and to decrease toxin biosynthesis (Peles et al., 2021). Examples of these bacterial biocontrol treatments are *Pseudomonas* spp., such as *P. fluorescens*, as studies indicated their potential against several fungi (Ganeshan and Manoj Kumar, 2005; Gull and Hafeez, 2012; Höfte, 2021). In our study, a new isolate that we identified to belong to the genus *Pseudomonas*, 20EI1, demonstrated a substantial effect on *A. flavus*, reducing or blocking its growth when both organisms were incubated together.

Competition between *A. flavus* and *Pseudomonas* 20EI1 for nutrients, including iron, might occur in the co-cultures. Both organisms could be producing siderophores to capture a limited amount of this element to survive, and eliminating the competing organism by starvation (Haas, 2012). In our study *Pseudomonas* 20EI1 eliminates or reduces the presence of *A. flavus* from the environment (Figure 1; Supplementary Figures S1, S2); perhaps a more efficient iron acquisition by the bacteria might starve *A. flavus* from this element, leading to a drastic decrease in its growth. On the other hand, during iron sufficiency, in iron-supplemented cultures, the fungus survives, while the bacterial presence decreases over time. In this case, where iron is not limited, other factors might be at play where the fungus is able to kill the bacteria, possibly by starvation of other limited nutrients or by involving antibiotic production. Chemical warfare between fungi and bacteria has been previously described, for instance, in a study by Spraker et al. (2018), a SM produced by *Fusarium fujikuroi* accumulates near the site of infection by the ralsolamycin-producing *R. solanacearum* GMI1000. Fungal growth is reduced by the bacteria, however the fungus produces several metabolites with antibacterial activity. Additionally, it is worth noting that when iron is in abundance in the bacterial monoculture, the bacterial biomass decreases (Figure 1C). It is possible that above certain levels, iron could become toxic for the bacteria (Andrews et al., 2003). The mechanism behind this has been investigated in *E. coli*; when an increase in intracellular free iron occurs, this causes an increase in the Fenton reaction, producing hydroxyl radicals and ultimately causing DNA damage (Touati, 2000).

In a recent study, *A. flavus* was co-cultured with a novel *Pseudomonas* spp. isolated from soil and sugarcane juice in Brazil (Rabiço et al., 2024). This study showed reduced growth of *A. flavus* due to volatile organic compounds (VOCs) by an estimated 31%. This decrease was less drastic compared to what we observed in our cultures using 20EI1. In another similar study, VOCs released by *Pseudomonas stutzeri* YM6 inhibited *A. flavus* growth (Gong et al., 2022). However, in that study only VOC in air were used, instead of co-cultures (Gong et al., 2022). Although we did not examine *Pseudomonas* 20EI1 VOCs here, it is possible that VOCs could contribute to some of the observed *A. flavus* growth inhibition. Future studies could provide additional insight into this possible mechanism.

Siderophores also present an important antifungal activity, for example in the case of *P. aeruginosa* against *A. niger*, *A. flavus*, *A. oryzae*, *F. oxysporum*, and *Sclerotium rolfsii* (Manwar et al., 2004). Interestingly, our *A. flavus* transcriptome analysis indicated alteration of iron-related genes by the bacteria. Specifically, 70 of 333 annotated genes were found differentially expressed in the co-culture, including fungal siderophore transporters and biosynthetic genes that were highly upregulated by the bacteria, consistent with *Pseudomonas* 20EI1 possibly reducing the available iron in the environment and showing the attempt of *A. flavus* to capture this metal. Specifically, expression of transporter genes, such as *mirB*, *mirD*, *sit1*, and *atrH*, as well as the biosynthesic genes *sidI*, *sidH*, *sidF*, and *sidD* of the TAFC siderophore were highly upregulated in the presence of 20EI1, in agreement with the increased need for iron capture. The addition of iron to the co-cultures reduced the expression of the TAFC-related genes in an equal but opposite direction. Very little is known about the effect of iron on *A. flavus*. Our study also showed that iron alone also influences the expression of iron-related genes in this fungus. These findings closely resemble those from the model yeast *Saccharomyces cerevisiae* growing in an environment with high iron concentration, where the fungus would not need to use these iron-related gene products to acquire additional iron and thus those genes are downregulated (Philpott, 2006). It is also likely that the mechanism in *A. flavus* is similar to that of *A. fumigatus* (Misslinger et al., 2021), where the siderophore-mediated iron acquisition (SIA) pathways is downregulated in the presence of high levels of extracellular iron.

In addition, during co-culture bacteria are strongly attached to the fungal hyphae when iron is not supplemented, as shown by our SEM images. Akocak et al. (2015) reported that *P. fluorescens* and *Bacillus* spp. present chitinolytic activity that causes hyphal leakage of *A. flavus*, however, in the case of *Pseudomonas* 20EI1 no signs of leakage where observed. When iron was supplemented, the bacterial population became reduced in the co-cultures, and attachment to fungal hyphae was not observed.

Importantly, our transcriptome analysis also revealed that *Pseudomonas* 20EI1 affects SM in *A. flavus*. More than 50% of the fungal genes in 13 BGCs are differentially expressed in the presence of *Pseudomonas* 20EI1, causing downregulation in most cases, for example, a reduced expression of AF genes, including the endogenous cluster regulators *aflR* and *aflS* (previous called *aflJ*) (Chang, 2003; Wang et al., 2022). Our results were concomitant with a drastic decrease in AFB_1_ production. Addition of iron to the co-culture presented a modulating opposite effect on gene expression and although AF production in these co-cultures with iron was still almost completely inhibited, growth of *A. flavus* was not affected, pointing to inhibition mechanisms unrelated to growth. Decrease of AF production has also been observed in *A. flavus* exposed to *P. fluorescens* across media (PDB and peanut medium) and on peanuts, where a 97.8, 99.4 and 55.8% reduction rate was observed, respectively (Yang et al., 2017). Interestingly, the levels of AFB_1_ were also reduced in the fungal culture alone with added iron. This is a novel role of iron in relationship with AF production in *A. flavus*, as current literature mostly focuses on the influence of dietary iron enhancing the effect of AFB_1_ in causing liver cancer (Asare et al., 2007). Wiemann et al. (2014) showed that the synthesis of specific SMs was largely influenced by the availability of iron in *A. fumigatus*, and its effect is mediated by the iron-dependent transcription factors SreA and HapX (Haas, 2012; Wiemann et al., 2014). The corresponding homologs in *A. flavus*, AFLA_132440 and AFLA_006720, have not been investigated in *A. flavus*. These genes were slightly upregulated in the presence of the bacteria. Whether they play a role in SM in *A. flavus* has not been determined. Although supplemented iron might also have contributed to the decrease in AF in the fungal-bacterial co-culture, it is likely that the bacteria also caused AF inhibition in the co-culture, as the reduction in AF was more dramatic in these cultures compared to those of the fungal culture plus iron in the absence of bacteria.

In addition to the effect of *Pseudomonas* 20EI1 on the expression of the AF BGC, the bacteria also downregulated the expression of genes in the CPA, KA and imizoquin BGCs. Downregulation of *A. flavus* BGCs by bacteria has been previously observed, for example by *P. megaterium*. In co-culture, this bacterium downregulated 19 of these clusters (Kong et al., 2014; Gupta et al., 2020), impacting AFB_1_, and also CPA production. CPA and KA are both metal-binding metabolites. The most common CPA toxin, α-CPA, is an inhibitor of mammalian Ca^2+^-ATPases disrupting intracellular calcium flux (Chang and Ehrlich, 2011), while a closely related analog also produced by *A. flavus*, ß-CPA, has been shown to bind iron and affect the growth of *Pseudomonas aeruginosa* (Guo et al., 2023). KA, usually utilized in food industry, also presents antibacterial activity (Mahmoud et al., 2024) and binds iron (Sudhir et al., 2005). Imizoquin has been shown to present a protective role against oxidative stress in *A. flavus* (Khalid et al., 2018). Of these three compounds, CPA and KA were detected in our chemical analysis, and coinciding with the downregulation of their respective BGCs, production of these compounds was dramatically decreased in *A. flavus* by the bacteria in an iron-independent manner—although iron caused an increase in the expression of genes in those BGCs in co-culture, it was not sufficient to establish CPA and KA production suggesting another bacterial mode of action preventing their production.

A different expression pattern was observed in co-culture with *Pseudomonas* 20EI1, where the expression of genes in the aspergillicin BGC increased, while addition of iron decreased their expression. Aspergillicins are siderophores produced by *A. flavus* (Greco et al., 2019). It is likely that under iron starvation caused by the bacteria in the culture, *A. flavus* increased expression of these genes in an attempt to capture iron, and in the cases of cultures with abundant iron those genes were found repressed to prevent further iron acquisition.

Interestingly, while examining the effect of *Pseudomonas* 20EI1 on global regulatory genes in *A. flavus*, our transcriptome data indicated that the putative C_2_H_2_ transcription factor gene, *mtfA*, was significantly upregulated in the presence of the bacteria. The *mtfA* gene was first characterized in the model fungus *A. nidulans* where it was demonstrated to control development and secondary metabolism (Ramamoorthy et al., 2013), including the expression of genes involved in the synthesis of sterigmatocystin, penicillin and terrequinone. In *A. flavus*, *mtfA* overexpression results in a drastic reduction or elimination of several secondary metabolites, including AFB₁ (Zhuang et al., 2016). This decrease in AF was also accompanied by a reduction in *aflR* expression. It is possible that the effect of *Pseudomonas* 20EI1 on *A. flavus* SM could be in part mediated by MtfA, as a response to the presence of the bacteria in the environment, resulting in alterations of the *A. flavus* SM profile.

## 5 Conclusion

In conclusion, we have established that *Pseudomonas* 20EI1 is a strong biocontrol that affects *A. flavus* growth and the expression of BGCs in this agriculturally important fungus, resulting in alterations in the production of SM, that includes a drastic reduction of AF, CPA and KA levels. Interestingly, the effect of 20EI1 on *A. flavus* occurs in an iron-dependent manner, affecting the expression of numerous fungal iron-related genes, including those encoding iron transporters and siderophore biosynthetic genes. It is likely that in the warfare of iron-related proteins, a more efficient iron sequestration by the bacteria starves *A. flavus*, a condition that is remediated by increasing the abundance of iron in the environment. Here we also show for the first time that iron itself reduces AF production in this fungus. Additionally, the presence of *Pseudomonas* 20EI1 induced the upregulation of the global regulatory gene *mtfA* in *A. flavus*, which could contribute to the observed decrease of SM production, including AF. Future studies will focus on the application of this promising biocontrol to crops in laboratory and agricultural settings with the goal of reducing the detrimental effects of *A. flavus* and possibly of other mycotoxigenic fungi.

## Data Availability

The dataset generated and analyzed during this study are available in the Sequence Read Archive (SRA), accessible at https://www.ncbi.nlm.nih.gov/bioproject/PRJNA1207321, Accession: PRJNA1207321.
