# Supplementary material for: An environmental isolate of Pseudomonas, 20EI1, reduces Aspergillus flavus growth in an iron-dependent manner and alters secondary metabolism
Source: Front Microbiol. 2025 Jan 20;15:1514950. doi: 10.3389/fmicb.2024.1514950 (PMC11788345; doi:10.3389/fmicb.2024.1514950)
Supplement: Supplementary file 5 [file Data_Sheet_5.PDF]

A Control B 20EI1

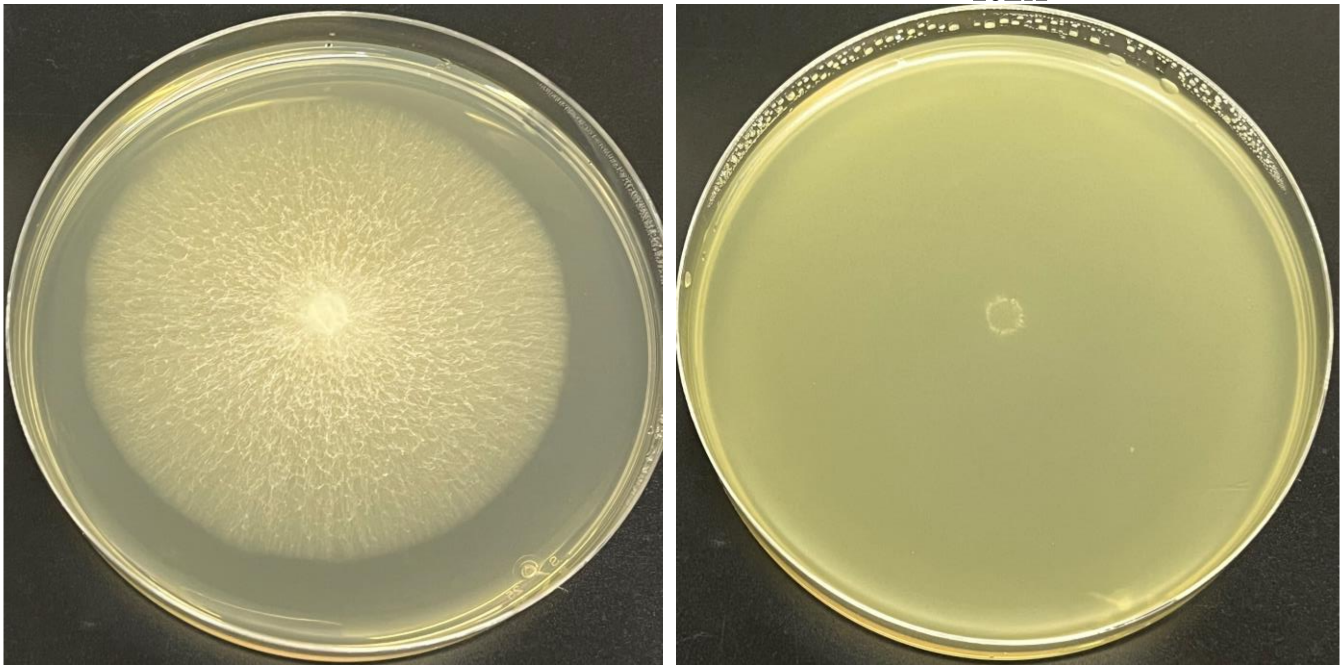

Supplementary Figure 1. Solid co-cultures of *Pseudomonas* 20EI1 and *Aspergillus flavus*. *A. flavus* with (B) and without (A) bacterial treatment grown at 30°C on PDA for 5 days in the dark.. Top agarose was inoculated with overnight bacterial culture and poured onto PDA plates. Fungal spores were point-inoculated at the center of the plate. The experiment was carried out in triplicate.

A Control B 20EI1 C *E. coli*

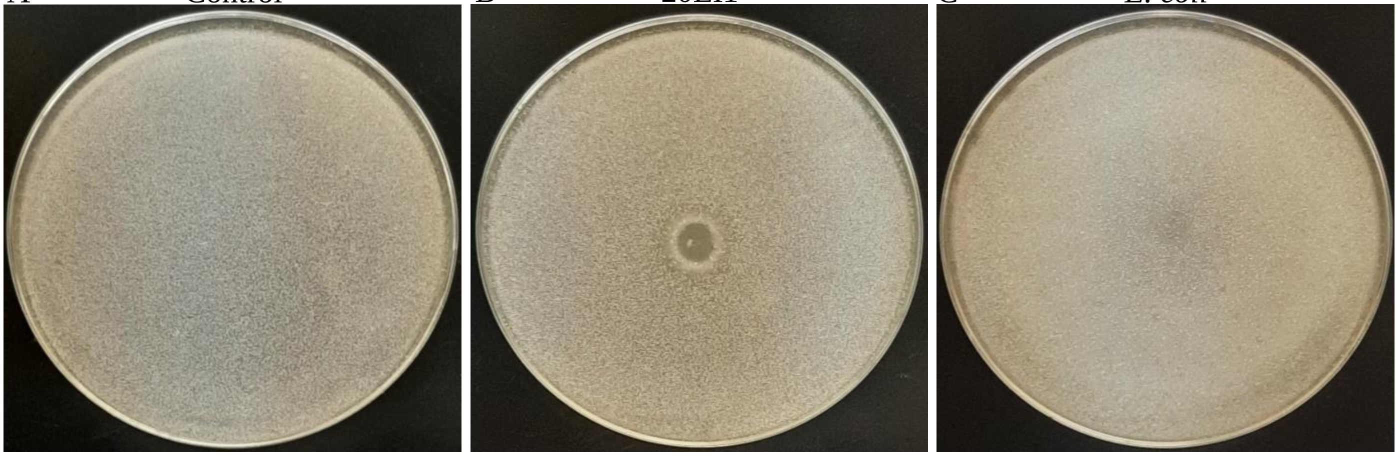

Supplementary Figure 2. Solid co-cultures of *Pseudomonas* 20EI1 and *Aspergillus flavus*. Spores of *A. flavus* were inoculated in the top agarose. *A. flavus* with (B, C) and without (A) bacterial treatment grown at 30°C on modified Czapeks Dox for 3 days in the dark. An overnight culture of 20EI1 (B) and *E. coli* (C), included as a control, were point-inoculated at the center of the plate. The experiment was carried out in triplicate.

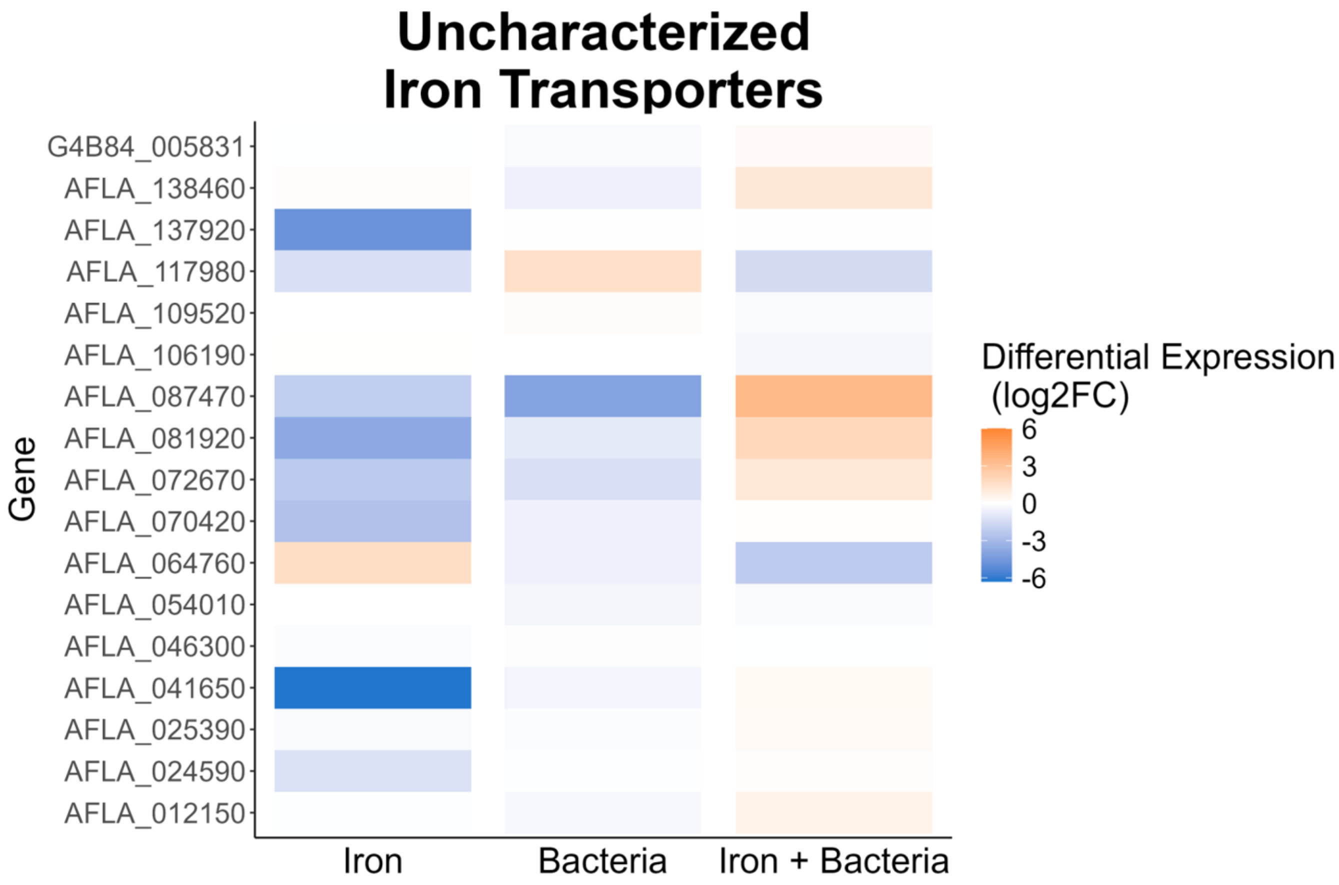

Supplementary Figure 3. Heatmaps showing differential expression of genes with functional annotations related to iron homeostasis in *A. flavus*. The conditions used to calculate differential expression are indicated along the x-axis. Color of the heatmap corresponds to the Log2FoldChange of the differential expression.
